# Round the Hospitals

**Published:** 1917-12-22

**Authors:** 


### ROUND THE HOSPITALS.

One notable fact which experience brings home to the observant is that when the pressure of increasing duties, and the claims for an ever-extending outlook of personal service, grow burdensome to weariness, then is the time of all times to uplift one's heart and lead one's fellows to further efforts in the great cause which every one of us is concerned in furthering in war-time. At the beginning of December it became demonstrably apparent that the spirit of Christmas was challenged and canvassed with the misplaced energy of a Bolo. The problem was to meet Bolo face to face, draw him out into the open and, with the aid of the sunlight, to dissolve him like the morning mist. We did our best in this emergency to secure this result, and our readers last week had interesting evidence that Bolo was not going to have it all his own way in our hospitals this year. To-day we have further evidence that the usual custom in regard to the Christmas festivities in the hospitals will be adhered to to the fullest extent possible. It is evident, therefore, that the sprite in question on Tuesday next will nowhere find a footing in our hospitals. Throughout the country the hospitals are everywhere giving Bolo the cold shoulder.

☛ Previous articles appeared December 8 and 15.

### A Moment of Leisure.

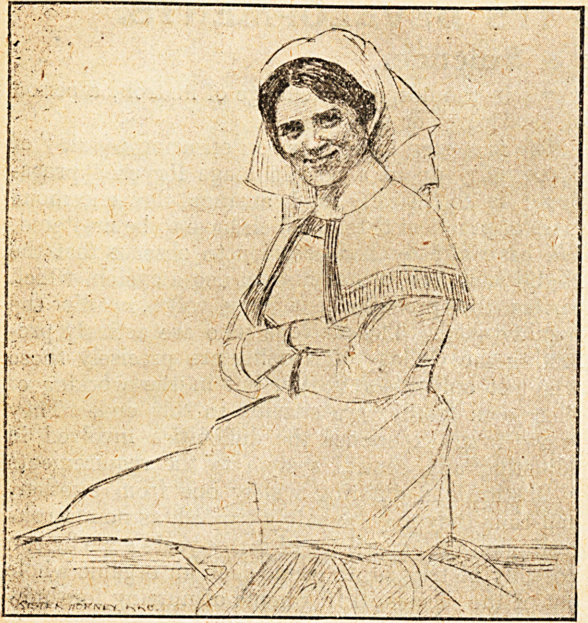

Being a Study. (By Corporal J. Hodgson Lobley.)

**ROUND THE HOSPITALS—*(continued)*.**

To-day we have the pleasure to announce that at St. Bartholomew's Hospital Christmas this year will be observed as usual. There will be a short special service with carols by the nursing staff in the Church of St. Bartholomew-the-Less. On Christmas morning the sisters will distribute gifts in their respective wards. The dinners will consist of good Christmas fare. From 3.30 to 6 p.m. entertainments will be held in the wards, several Pierrot troupes and other artistes having most kindly promised to assist the residents in making them a memorable success.

Guy's Hospital has for decades been famed throughout the country for the hearty Christmas festivities which its patients have been privileged to enjoy. Christmas there this year will be a season of real, if restrained, rejoicing. The wards will be cheerful with decorations, although the coloured electric lights, costing much labour in wiring, may be absent. During Christmas week concerts and entertainments by the residents and their friends will be given, and of clothing or other useful articles every patient will have some. The sisters, too, will be spared personal outlay to a ruinous extent by the provision of a Christmas Fund, of which each will have her proper share. Many years ago the yielders of personal service, with the cordial co-operation of the authorities, instituted The Out-Patient Tea, and this year, as usual, it will prove once more an important event for the poor children of the neighbourhood. At two o'clock on Christmas Day the seven hundred small guests, full of expectation, will assemble in the out-patient hall, where the festivities will include a nigger entertainment by the residents, a famous Christmas Tree, and a gift for every child. Owing to the action of the Food Controller the packets of tea, which in years of peace were taken home by the guests as "Mother's share," will this year fail, but a suitable and acceptable substitute has been found. There is also a new feature this year in connection with the maternity charity, and a Christmas dinner will be provided for about a dozen carefully selected deserving cases, who in addition will receive some coal, or a goodly gift of money to defray the cost of gas. Well done, Guy's!

The family at the London Hospital in the Whitechapel Road will be larger than ever on Christmas Day this year, and it shows a commendable desire to lay Bolo low that arrangements should have been made to provide that Christmas at that institution will this year be upheld and nurtured. The difficulties have been immense, for the housemen or residents there now continue in their appointments for so short a time that there has been no opportunity to hold the necessary rehearsals to provide the troupes who have made a practice of going round the wards carrying with them innocent amusement, hilarity, and stimulation. The children, however, as their Majesties have endeavoured to ensure throughout the metropolitan hospitals, will be cheered by the presence of Mr. Punch and Judy. Father Christmas will also make his welcome presence realised by the inmates, and a few friends, who are friends indeed, are voluntarily devoting their time on Christmas Day to singing and reciting in the wards. The children of Whitechapel, to the number of 700 or 800, many of them cripples, are to have this year a Christmas party on December 28, when four Christmas Trees will gladden their hearts and secure a present for everybody, in addition to the toys with which the children will be provided by the kind and generous help of the *Daily Mail*.

We are informed by the authorities of the National Hospital for the Paralysed and Epileptic that for some time the question of turkey on Christmas Day at this institution hung in the balance, but it was finally decided by some kind friends generously providing the extra money. Many managers will rejoice that by prudent buying the pudding for Christmas will very nearly approach its usual standard of excellence there. Though several features of former years may necessarily be absent from the Christmas programme, the 170 patients, 70 of whom are soldiers "suffering from blindness, mutism, deafness, and the results of nerve injury and nerve diseases," will, the authorities guarantee, have a good time.

An attractive programme is in store at the General Hospital, Birmingham, beginning with carols to be sung in the wards by the night nurses before going on duty on Christmas Eve. On Christmas morning there will be Holy Communion at 6 o'clock and 8 o'clock. At 9.45 the Lord Mayor and Lady

**ROUND THE HOSPITALS—(*continued*).**

Mayoress will visit the wards, matins and a sermon follow, and then comes the patients' dinner. The afternoon will be given up to Father Christmas, who, with a troupe of entertainers in fancy dress, will visit the various wards, the day's entertainment winding up with a concert in Accident Ward No. 2. Entertainments of various kinds will be given during the week, including the Christmas Tree in the Children's Wards. On New Year's Day a concert and supper for soldiers in Ward 7 is being anticipated with genuine pleasure. Jaffray Hospital, too, will have its Christmas Tree on Christmas Day.

---

We hope with the aid of our contemporaries in the lay press, that the evidence we have here given of the successful laying low and annihilation of Bolo throughout our hospitals may be brought home to the hearts and consciences of the residents throughout London, and that many of them may be awakened to take an interest in the sufferers within the hospitals on Christmas Day. Can anyone who is privileged to be a governor of a hospital fail this year to render the personal service of communicating with the house governor, the matron, or the secretary of some hospital known to each, with the view of providing such additional aid to the joyous celebration of Christmas Day in the direction which they have individually found to be most inspiring and helpful to themselves?

It has always been our custom not to publish items of personal news, or, indeed, any items, until we have evidence that the time has arrived when publication would do good, free from any risk of an opposite result. We now have much pleasure in announcing that Miss Sidney Browne, R.R.C., Matron-in-Chief of the Territorial Force Nursing Service, has been appointed a member of the Council of the College of Nursing. Experience teaches that a worker whose results prove most successful, and have become recognised as of the highest value, avoids the limelight and is seldom heard of. There is not a single member of the Territorial Force Nursing Service, we are confident, who does not render loyal, grateful, and loving homage to their chief, Miss Sidney Browne. All of them will welcome her appointment as a member of the Council of the College of Nursing, Limited, as a matter of justice to herself and of importance to their Service and to British nursing.

---

Last week we published the result of the special general meeting of the Royal British Nurses' Association held on the 12th instant. At that meeting a resolution of sympathy with Princess Christian in her bereavement was carried by all present rising from their seats. It was announced, amidst applause, that Princess Arthur of Connaught had kindly consented to become a Vice-President of the Association. The Chairman, Dr. Kenneth Stewart, read a letter from H.R.H. Princess Christian, the President, in which H.R.H. pointed out the object of summoning the meeting, and

☛ Previous articles appeared October 27 and December 1, 1917.

**ROUND THE HOSPITALS**—(*continued*).

explained that the Honorary Medical Secretary would report on the negotiations between the College of Nursing and the R.B.N.A. The letter proceeded:—While H.R.H. had every confidence in the Council, she felt it was only right that a report of what had taken place should be laid before a general meeting, so as to allow an opportunity for discussion. She knew that the nurses on the Council gave the matter very careful consideration, and H.R.H. felt that the proper and constitutional course for her to take was to leave a decision so vitally affecting their interests in their own hands. It was important that all members should be made fully acquainted with all that had happened, so that the Council might be assured that they possessed the full trust and confidence of the members. Her one aim and object had always been, and always would be, to safeguard and advance the interests of the nursing profession by every means in her power; and she hoped that the decision which had been come to would be conducive in every respect to that end.

---

A nursing paper noted for the moderation of its language and the prodigality of its tarradilloes is still unhappy. Its latest worry is the increasing success of the College of Nursing, Limited. It has made the College of Nursing its stalking-horse and endless topic for the supply of items, which have kept its editor in copy for months. In its latest issue, with an ingratitude born of boredom, in sad contrast to the excellence of its deportment and perfect manners, which cannot fail to amuse the nursing profession here and in the United States, it denominates the College of Nursing "this tiresome theme." May this lapse be accepted as a sign of repentance, of exhaustion, or what?

---

A movement is on foot in Dublin, of which Miss Minnie Ross, 33 Molesworth Street, is honorary secretary, to raise a fund in memory of the late Miss Bradshaw, who was for ten years secretary to Lady Dudley's Nursing Scheme for the establishment of nurses in the poorest districts of Ireland. For fourteen years Miss Bradshaw had been organising secretary of the Irish Central Bureau for the Training and Employment of Women, and before that assistant secretary of the South African Colonisation Society. Miss Bradshaw's health was never very good, and, as her infirmity increased, she continued to put all her available energy into the work until her death in August last at the early age of forty-five. She won for herself a warm place in the hearts of the Irish people, and her numerous friends and fellow-workers, realising the debt they owe Miss Bradshaw for her inspiring example of exacting work carried out with great ability and thoroughness, coupled with unfailing courage and cheerfulness under most adverse circumstances, are wishful to raise a memorial fund to assist many younger gentlewomen, whose lot it is to earn their living, when they are in danger of falling out of the ranks from illness due to over-work or other causes, by affording them the rest and medical treatment necessary to restore them to health. The Countess of Mayo is the president, and many members of the Committee for whom Miss Bradshaw worked have become vice-presidents, facts which guarantee the soundness of the administration of the Fund. At the moment all contributions are being accumulated and invested to form a permanent fund; but much larger and more general support by subscriptions and donations is still required before the income available will be enough to enable the Committee to make grants. Every information can be obtained from the Honorary Secretary, Myrrha Bradshaw Memorial Fund, 33 Molesworth Street, Dublin, by whom contributions of any amount will be gratefully received.

---

☛ It is our invariable practice to pay for all items of news which we may publish. The minimum payment is 5s. Paragraphs of about twenty lines in length—that is to say, of 150 words or less—are preferred. Contributions should be addressed to The Editor, The Hospital, 28 and 29 Southampton Street, Strand, London, W.C. 2, and, to be in time for the current week's issue, should reach him by the first post on Mondays.

For Nursing Affairs in Ireland, see p. 256.

